# Supplementary figures and images for: A prospective, multicenter, post-marketing observational study to measure the quality of life of HCV genotype 1 infected, treatment naïve patients suffering from fatigue and receiving 3D regimen: The HEMATITE study
Source: PLoS One. 2020 Nov 4;15(11):e0241267. doi: 10.1371/journal.pone.0241267 (PMC7641439; doi:10.1371/journal.pone.0241267)

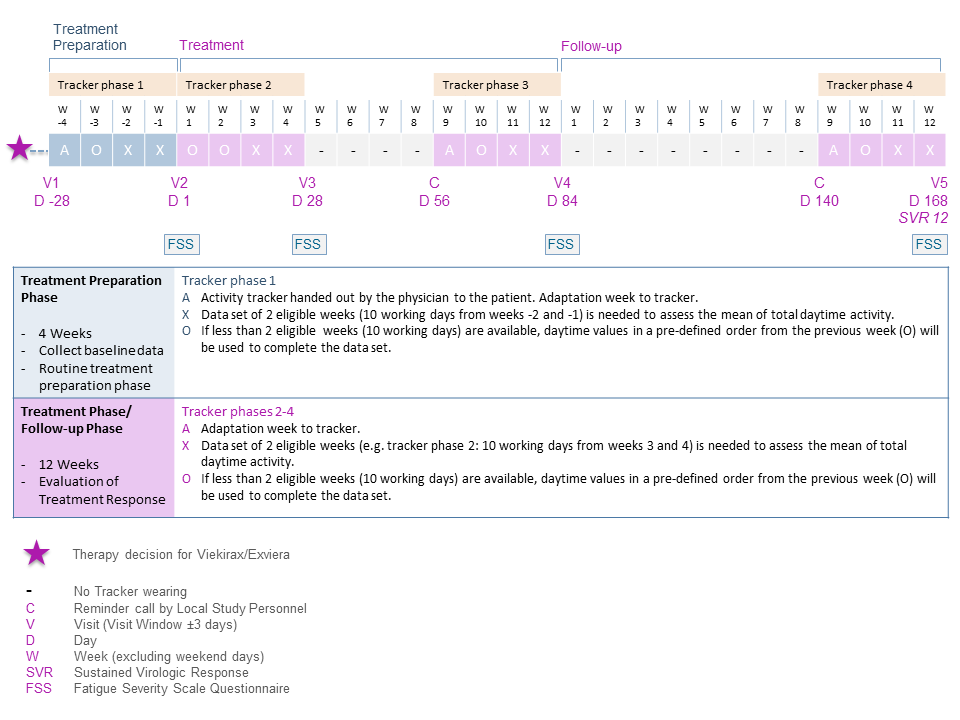

Supplement: S1 Fig — The 28 week observation period was comprised of 4 weeks preparation, 12 weeks treatment and 12 weeks follow-up. Screening took place at Day -28 (Study Visit 1). Fatigue was assessed by FSS questionnaire on Days 1, 28, 84 and 168 (Study Visits 2, 3, 4 and 5, respectively). Daytime physical activity and sleep efficiency were assessed by activity tracker during 4 x 4 week tracker phases. Baseline data were collected in tracker phase 1. A data set of 2 eligible weeks (10 working days) was used to assess daytime physical activity and sleep efficiency from tracker phases 1, 2, 3 and 4. SVR12 was determined on Day 168 (12 weeks post-treatment). (TIF) [file pone.0241267.s002.TIF]

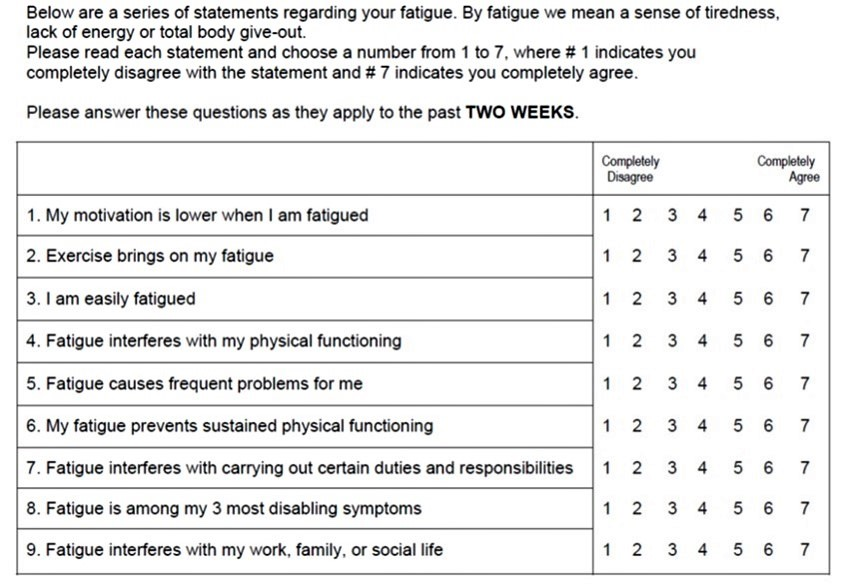

Supplement: S2 Fig — Taken from Krupp LB, LaRocca NG, Muir-Nash J, Steinberg AD. The fatigue severity scale. Application to patients with multiple sclerosis and systemic lupus erythematosus. Arch Neurol 1989;46:1121–1123. (TIF) [file pone.0241267.s003.tif]

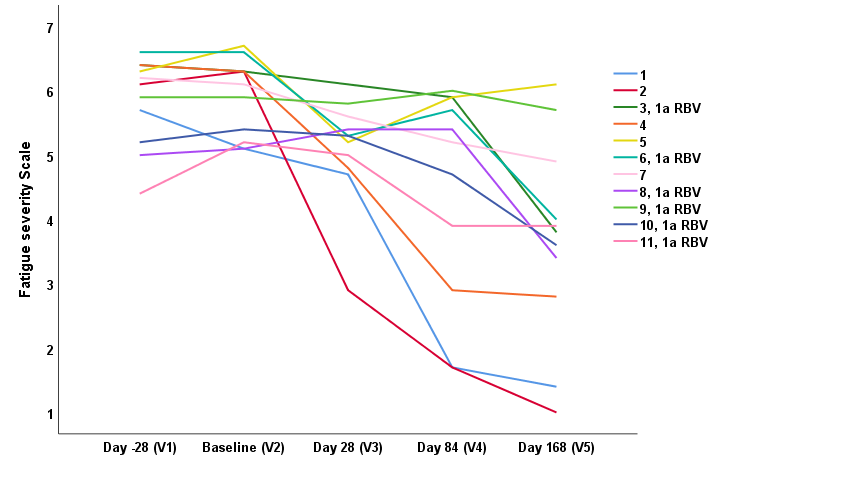

Supplement: S3 Fig — (TIF) [file pone.0241267.s004.tif]
