# Supplementary material for: A prospective, multicenter, post-marketing observational study to measure the quality of life of HCV genotype 1 infected, treatment naïve patients suffering from fatigue and receiving 3D regimen: The HEMATITE study
Source: PLoS One. 2020 Nov 4;15(11):e0241267. doi: 10.1371/journal.pone.0241267 (PMC7641439; doi:10.1371/journal.pone.0241267)
Supplement: S1 Table — (DOC) [file pone.0241267.s005.doc]

**S1 Table Possible predictors for the outcomes, sdITT (n = 37)**

| **Factor** | | **Category** | **Change V2 – V5 (95% CI)** | **p value / part. η^2^** | |
| --- | --- | --- | --- | --- | --- |
|  | |  |  | **univariate** | **multivariate** |
| ***Mean daytime physical activity*** | | | | | |
| **Age class** | | |  |  |  |
|  | ≤ 50 years (n = 13) | | -152306.5 (-394612.5 – 89999.6) | 0.751 / 0.005 | 0.840 / 0.002 |
|  | > 50 years (n = 13) | | -64226.0 (-185448.9 – 56996.8) |  |  |
| **Sex** | | |  |  |  |
|  | Male (n = 10) | | -150195.7 (-356002.1 – 55610.7) | 0.927 / <0.001 | 0.941 / <0.001 |
|  | Female (n = 16) | | -82060.4 (-260877.2 – 96756.4) |  |  |
| **Fibrosis** | | |  |  |  |
|  | Stage 0 (n = 10) | | -58132.46 (-178248.2 – 61983.3) | 0.676 / 0.008 | 0.676 / 0.010 |
|  | Stage ≥ 1 (n = 16) | | -139599.9 (-342185.6 – 62985.8) |  |  |
| **HCV genotype** | | |  |  |  |
|  | GT 1a (n = 16) | | -66509.1 (-189850.3 – 56832.1) | 0.074 / 0.138 | 0.223 / 0.081 |
|  | GT 1b (n = 10) | | -1750777 (-475421.4 – 125266.1) |  |  |
| **Ribavirin** | | |  |  |  |
|  | Yes (n = 14) | | -26941.2 (-153441.8 – 99559.4) | 0.266 / 0.056 | 0.658 / 0.011 |
|  | No (n = 12) | | -203145.4 (-448329.9 – 42039.0) |  |  |

**Continued S1 Table Possible predictors for the outcomes, sdITT (n = 37)**

| **Factor** | | **Category** | **Change V2 – V5 (95% CI)** | **p value / part. η^2^** | | |
| --- | --- | --- | --- | --- | --- | --- |
|  | |  |  | **univariate** | | **multivariate** |
| ***Sleep efficiency*** | | | | | | |
| **Age class** | | |  |  | |  |
|  | ≤ 50 years (n = 13) | | -0.245 (-2.281 – 1.791) | 0.496 / 0.021 | | 0.257 / 0.071 |
|  | > 50 years (n = 13) | | 1.466 (-0.394 – 3.327) |  | |  |
| **Sex** | | |  |  | |  |
|  | Male (n = 10) | | 0.772 (-2.154 – 3.699) | 0.055 / 0.157 | | 0.066 / 0.175 |
|  | Female (n = 16) | | 0.510 (-0.985 – 2.004) |  | |  |
| **Fibrosis** | | |  |  | |  |
|  | Stage 0 (n = 10) | | -0.153 (-2.524 – 2.219) | 0.198 / 0.074 | | 0.200 / 0.089 |
|  | Stage ≥ 1 (n = 16) | | 1.088 (-0.656 – 2.832) |  | |  |
| **HCV genotype** | | |  |  | |  |
|  | GT 1a (n = 16) | | -0.573 (-2.285 – 1.140) | 0.222 / 0.067 | | 0.424 / 0.036 |
|  | GT 1b (n = 10) | | 2.504 (0.722 – 4.285) |  | |  |
| **Ribavirin** | | |  |  | |  |
|  | Yes (n = 14) | | -0.280 (-2.210 – 1.651) | 0.639 / 0.010 | | 0.849 / 0.002 |
|  | No (n = 12) | | 1.649 (-0.263 – 3.561) |  | |  |
| ***FSS*** | | | | | | |
| **Age class** | | |  |  |  | |
|  | ≤ 50 years (n = 19) | | 2.619 (1.945 – 3.293) | 0.176 / 0.055 | 0.202 / 0.056 | |
|  | > 50 years (n = 18) | | 2.576 (1.647 – 3.506) |  |  | |
| **Sex** | | |  |  |  | |
|  | Male (n = 12) | | 2.087 (1.145 – 3.029) | 0.844 / 0.001 | 0.699 / 0.005 | |
|  | Female (n = 25) | | 2.844 (2.160 – 3.527) |  |  | |
| **Fibrosis** | | |  |  |  | |
|  | Stage 0 (n = 14) | | 2.260 (1.284 – 3.236) | 0.415 / 0.020 | 0.227 / 0.050 | |
|  | Stage ≥ 1 (n = 23) | | 2.804 (2.121 – 3.487) |  |  | |
| **HCV genotype** | | |  |  |  | |
|  | GT 1a (n = 21) | | 2.623 (1.949 – 3.298) | 0.335 / 0.028 | 0.616 / 0.009 | |
|  | GT 1b (n = 16) | | 2.565 (1.586 – 3.544) |  |  | |
| **Ribavirin** | | |  |  |  | |
|  | Yes (n = 19) | | 2.655 (1.975 – 3.334) | 0.267 / 0.037 | 0.915 / <0.001 | |
|  | No (n = 18) | |  |  |  | |
