# Supplementary material for: A prospective, multicenter, post-marketing observational study to measure the quality of life of HCV genotype 1 infected, treatment naïve patients suffering from fatigue and receiving 3D regimen: The HEMATITE study
Source: PLoS One. 2020 Nov 4;15(11):e0241267. doi: 10.1371/journal.pone.0241267 (PMC7641439; doi:10.1371/journal.pone.0241267)
Supplement: S2 Table — (DOC) [file pone.0241267.s006.doc]

**S2 Table Possible predictors for the outcomes, mITT (n = 24)**

| **Factor** | | **Category** | **Change V2 – V5 (95% CI)** | **p value / part. η^2^** | |
| --- | --- | --- | --- | --- | --- |
|  | |  |  | **univariate** | **multivariate** |
| ***Mean daytime physical activity*** | | | | | |
| **Age class** | | |  |  |  |
|  | ≤ 50 years (n = 12) | | -144508.7 (-409948.9 – 120931.4) | 0.751 / 0.005 | 0.840 / 0.002 |
|  | > 50 years (n = 12) | | -52237.3 (-182234.1 – 77759.6) |  |  |
| **Sex** | | |  |  |  |
|  | Male (n = 8) | | -130998.3 (-400879.5 – 138883.0) | 0.927 / < 0.001 | 0.941 / < 0.001 |
|  | Female (n = 16) | | -82060-4 (-260877.2 – 96756.4) |  |  |
| **Fibrosis** | | |  |  |  |
|  | Stage 0 (n = 8) | | -15919.2 (-150631.9 – 118793.4) | 0.676 / 0.008 | 0.676 / 0.010 |
|  | Stage ≥ 1 (n = 16) | | -139599.9 (-342185.6 – 62985.8) |  |  |
| **HCV genotype** | | |  |  |  |
|  | GT 1a (n = 14) | | -43583.9 (-181719.6 – 94551.7) | 0.074 / 0.138 | 0.223 / 0.081 |
|  | GT 1b (n = 10) | | -175077.7 (-475421.4 – 125266.1) |  |  |
| **Ribavirin** | | |  |  |  |
|  | Yes (n = 12) | | 6399.5 (-133057.9 – 145856.8) | 0.266 / 0.056 | 0.658 / 0.011 |
|  | No (n = 12) | | -203145.44 (-448329.9 – 42039.0) |  |  |
| ***Sleep efficiency*** | | | | | |
| **Age class** | | |  |  |  |
|  | ≤ 50 years (n = 12) | | -0.081 (-2.282 – 2.121) | 0.496 / 0.021 | 0.257 / 0.071 |
|  | > 50 years (n = 12) | | 1.563 (-0.467 – 3.593) |  |  |
| **Sex** | | |  |  |  |
|  | Male (n = 8) | | 1.204 (-2.534 – 4.942) | 0.055 / 0.157 | 0.066 / 0.175 |
|  | Female (n = 16) | | 0.510 (-0.985 – 2.004) |  |  |
| **Fibrosis** | | |  |  |  |
|  | Stage 0 (n = 8) | | 0.048 (-3.018 – 3.114) | 0.198 / 0.074 | 0.200 / 0.089 |
|  | Stage ≥ 1 (n = 16) | | 1.088 (-0.656 – 2.832) |  |  |
| **HCV genotype** | | |  |  |  |
|  | GT 1a (n = 14) | | -0.518 (-2.488 – 1.453) | 0.222 / 0.067 | 0.424 / 0.036 |
|  | GT 1b (n = 10) | | 2.504 (0.722 – 4.286) |  |  |
| **Ribavirin** | | |  |  |  |
|  | Yes (n = 12) | | -0.167 (-2.443 – 2.109) | 0.639 / 0.010 | 0.849 / 0.002 |
|  | No (n = 12) | | 1.649 (-0.263 – 3.561) |  |  |

**Continued S2 Table Possible predictors for** the outcomes, mITT (n = 24)

| **Factor** | | **Category** | **Change V2 – V5 (95% CI)** | **p value / part. η^2^** | |
| --- | --- | --- | --- | --- | --- |
|  | |  |  | **univariate** | **multivariate** |
| ***FSS*** | | | | | |
| **Age class** | | |  |  |  |
|  | ≤ 50 years (n = 12) | | 2.556 (1.697 – 3.415) | 0.060 / 0.166 | 0.072 / 0.189 |
|  | > 50 years (n = 12) | | 3.078 (2.011 – 4.146) |  |  |
| **Sex** | | |  |  |  |
|  | Male (n = 8) | | 2.294 (0.943 – 3.645) | 0.909 / 0.001 | 0.614 / 0.016 |
|  | Female (n = 16) | | 3.079 (2.310 – 3.848) |  |  |
| **Fibrosis** | | |  |  |  |
|  | Stage 0 (n = 8) | | 2.703 (1.485 – 3.920) | 0.589 / 0.015 | 0.423 / 0.041 |
|  | Stage ≥ 1 (n = 16) | | 2.874 (2.029 – 3.720) |  |  |
| **HCV genotype** | | |  |  |  |
|  | GT 1a (n = 14) | | 2.789 (2.018 – 3.560) | 0.222 / 0.074 | 0.350 / 0.055 |
|  | GT 1b (n = 10) | | 2.857 (1.560 – 4.154) |  |  |
| **Ribavirin** | | |  |  |  |
|  | Yes (n = 12) | | 2.866 (2.161 – 3.601) | 0.200 / 0.081 | 0.663 / 0.012 |
|  | No (n = 12) | | 2.768 (1.587 – 3.950) |  |  |
