# Supplementary material for: A prospective, multicenter, post-marketing observational study to measure the quality of life of HCV genotype 1 infected, treatment naïve patients suffering from fatigue and receiving 3D regimen: The HEMATITE study
Source: PLoS One. 2020 Nov 4;15(11):e0241267. doi: 10.1371/journal.pone.0241267 (PMC7641439; doi:10.1371/journal.pone.0241267)
Supplement: S1 File — (PDF) [file pone.0241267.s007.pdf]

AbbVie AG (AbbVie)

Post Marketing Observational Study

Protocol P16-272

# Quality of Life measurement using wrist actigraphy in HCV genotype 1 infected, treatment naïve patients suffering from fatigue and receiving ombitasvir, paritaprevir, and ritonavir tablets and dasabuvir tablets (Viekirax®/Exviera®; 3D regimen): The HEMATITE Study

| | |
|---|---|
| Product Name: | ombitasvir, paritaprevir, and ritonavir tablets and dasabuvir tablets (Viekirax®/Exviera®; 3D regimen) |
| Type of Study: | Observational Study |
| Status of Document: | V1.0 |
| Date: | 20 September 2016 |
| Sponsor | AbbVie AG, Switzerland |

**This study will be conducted in compliance with this protocol.**
**Confidential Information. No use or disclosure outside AbbVie is permitted without prior written**

| | |
|---|---|
| **Title** | Quality of life measurement using wrist actigraphy in HCV genotype 1 infected, treatment naïve patients suffering from fatigue and receiving ombitasvir, paritaprevir, and ritonavir tablets and dasabuvir tablets (Viekirax®/Exviera®; 3D regimen): The HEMATITE Study |
| **Protocol Version Identifier** | P16-272 |
| **Date of Last Version of Protocol** | 20 September 2016 |
| **EU PAS Register Number** | Not Applicable |
| **Active Substance** | Ombitasvir, paritaprevir, and ritonavir tablets; dasabuvir tablets (Viekirax®/Exviera®,' 3D regimen) |
| **Medicinal Product** | Ombitasvir, paritaprevir, and ritonavir tablets; dasabuvir tablets (Viekirax®/Exviera®,' 3D regimen) |
| **Product Reference** | Not Applicable |
| **Procedure Number** | Not Applicable |
| **Marketing Authorization Holder(s)** | AbbVie AG Schweiz<br>Neuhofstrasse 23<br>CH - 6341 Baar |
| **Joint PASS** | Not Applicable |
| **Research Question and Objectives** | To observe the impact of the 3D regimen on total daytime physical activity in Hepatitis C Virus (HCV) positive patients in a real-world setting.<br>Objectives:<br>• To observe changes in physical activity (assessed by means of an electronic wrist-worn activity tracker) in patients with newly initiated 3D regimen before, during and after treatment,<br>• To correlate subjective fatigue (assessed by means of the validated FSS (Fatigue Severity Scale) questionnaire) and physical activity,<br>• To observe the proportion of patients achieving sustained virologic response 12 weeks after end of treatment with 3D (SVR12). |

| Country(-ies) of Study | Switzerland |
| --- | --- |
| **Authors** | **AbbVie AG Schweiz**<br>Ralph Torgler, PhD<br>Lisa Ruckstuhl, MSc<br>Neuhofstrasse 23<br>CH - 6341 Baar |

## Marketing Authorization Holder(s)

| Marketing Authorization Holder(s) | AbbVie AG Schweiz<br>Neuhofstrasse 23<br>CH - 6341 Baar |
| --- | --- |
| **MAH Contact Person** | Not applicable |

# 1.0 Table of Contents


1.0 Table of Contents ........ 4
2.0 Abbreviations ........ 6
3.0 Responsible Parties ........ 7
4.0 Abstract ........ 8
5.0 Amendments and Updates ........ 13
6.0 Milestones ........ 13
7.0 Background and Rationale ........ 13
8.0 Research Question and Objectives ........ 15
8.1 Research Question ........ 15
8.2 Objectives ........ 15
8.2.1 Primary Objective ........ 15
8.2.2 Secondary Objectives ........ 15
9.0 Research Methods ........ 15
9.1 Study Design ........ 15
9.1.1 Description of Activities ........ 17
9.1.2 Description of Visits ........ 20
9.1.3 Product Supply ........ 21
9.1.4 Termination Criteria ........ 21
9.2 Setting ........ 22
9.2.1 Target Population ........ 22
9.2.2 Study Duration ........ 23
9.2.3 Investigator Selection Criteria ........ 23
9.3 Variables ........ 23
9.3.1 Primary Variable ........ 23
9.3.2 Secondary Variables ........ 24
9.4 Data Sources ........ 24
9.5 Study Size ........ 24
9.6 Data Management ........ 25
9.7 Data Analysis ........ 25
9.8 Quality Control ........ 26

| | | |
|---|---|---|
| 9.9 | Limitations of the Research Methods | 27 |
| 9.10 | Other Aspects | 28 |
| 10.0 | Protection of Human Subjects | 28 |
| 11.0 | Management and Reporting of Complaints | 28 |
| 11.1 | Medical Complaints | 29 |
| 11.1.1 | Adverse Event Definition and Serious Adverse Event Categories | 29 |
| 11.1.2 | Severity | 31 |
| 11.1.3 | Relationship to Pharmaceutical Product | 31 |
| 11.1.4 | Serious-, non Serious Adverse Event and Pregnancy Collection Period | 32 |
| 11.1.5 | Adverse Event reporting | 32 |
| 11.1.6 | Serious Adverse Event Reporting | 32 |
| 11.1.7 | Pregnancy Reporting | 33 |
| 11.2 | Product Complaint | 33 |
| 11.2.1 | Definition | 33 |
| 11.2.2 | Reporting | 34 |
| 12.0 | Plans for Disseminating and Communicating Study Results | 34 |
| 13.0 | References | 36 |
| Annex 1. | ActiGraph GT9X Link [26] | 39 |
| Annex 2. | Fatigue Severity Scale (FSS) [38] | 41 |
| Annex 3. | ICD-10 Version:2016 [41] | 42 |
| Annex 4. | List of Protocol Signatories | 43 |
| Annex 5. | Requirements for non-interventional studies per local laws and regulations | 44 |

## 2.0 Abbreviations

| | |
|---|---|
| 3D regimen | Ombitasvir, Paritaprevir, and Ritonavir tablets (Viekirax®); Dasabuvir tablets (Exviera®) |
| AASLD | American Association for the Study of Liver Diseases |
| AE | Adverse Event |
| BMI | Body Mass Index |
| CA | Competent Authorities |
| CHCV | Chronic Hepatitis C infection, defined as the presence of detectable viral replication for at least six months. |
| CNS | Central Nervous System |
| CRF | Case Report Form |
| CRO | Contract Research Organization |
| EASL | European Association for the Study of the Liver |
| EC | Ethics Committee |
| EMA | European Medicines Agency |
| EOT | End of Treatment |
| FSS | Fatigue Severity Scale |
| HCV | Hepatitis C Virus |
| ICMJE | International Committee of Medical Editors |
| LSLV | Last Subject Last Visit |
| MAH | Marketing Authorization Holder |
| PMOS | Post Marketing Observational Study |
| QoL | Quality of Life |
| RBV | Ribavirin |
| RNA | Ribonucleic acid |
| SAE | Serious Adverse Event |
| SAP | Statistical Analysis Plan |
| SmPC | Summary of Product Characteristics |
| SVR | Sustained Virologic Response |
| SVR12 | Sustained Virologic Response at12 weeks after EOT |
| V | Visit |
| WHO | World Health Organization |

# 3.0 Responsible Parties

**Study Designated Physician (SDP):**

- Marianne Saurenmann, MD
  Clinical Operations Manager
  AbbVie AG Schweiz
  Neuhofstrasse 23, CH-6341 Baar
  Office: +41 41 399 16 28
  Mobile: +41 79 474 23 58

**Protocol Authors:**

- Ralph Torgler, PhD
  Medical Manager HCV
  AbbVie AG Schweiz
  Neuhofstrasse 23, CH-6341 Baar
  Office: +41 41 399 15 40
  Mobile: +41 79 334 56 33

- Lisa Ruckstuhl, MSc
  Medical Advisor HCV
  AbbVie AG Schweiz
  Neuhofstrasse 23, CH-6341 Baar
  Office: +41 41 399 16 21
  Mobile: +41 79 334 58 56

**Pharmacovigilance AbbVie AG Schweiz:**

Phone: +41 41 399 16 09
Fax: +41 41 399 16 67
pharmacovigilance.ch@abbvie.com

**Quality Assurance AbbVie AG Schweiz:**

Phone: +41 41 399 16 98
Fax: +41 41 399 16 68
complaints.ch@abbvie.com

**Vendor:**

- GKM
  Gesellschaft für Therapieforschung mbH
  Lessingstrasse 14
  D-80336 München
  Germany

Contact details and a list of investigators will be kept at AbbVie and will be available upon request.

# 4.0 Abstract

**Title:** Quality of life measurement using wrist actigraphy in HCV genotype 1 infected, treatment naïve patients suffering from fatigue and receiving ombitasvir, paritaprevir, and ritonavir tablets and dasabuvir tablets (Viekirax®/Exviera®; 3D regimen): The HEMATITE Study

**Background and Rationale:**

Physical and mental fatigue is the most common symptom reported by patients with Hepatitis C Virus (HCV), which highly impacts their overall quality of life. AASLD (American Association for the Study of Liver Diseases ) and EASL (European Association for the Study of the Liver) guidelines therefore rate the treatment of HCV patients suffering from debilitating fatigue as a high priority. This cardinal symptom presents regardless of the stage of liver fibrosis and is difficult to quantify objectively. Similar to other potential reasons for physical fatigue, such as hepatic encephalopathy, increasing evidence suggests a direct viral impact on the central nervous system (CNS). Data demonstrating a longitudinal change of debilitating physical fatigue and increased daytime physical activity upon treatment with 3D regimen are missing to date and are anticipated by the Swiss scientific HCV community [1, 2].

The rationale for this observational study is to observe the impact of therapy with 3D regimen on physical activity of HCV patients suffering from debilitating fatigue. Furthermore, this study supports the Swiss Hepatitis Strategy, which seeks for the elimination of viral hepatitis in Switzerland within the next 15 years by creating awareness for patients with extrahepatic manifestations [3].

**Research Question and Objectives:**

Does successful treatment with 3D regimen increase total daytime physical activity and reduce fatigue in HCV-positive patients?

Primary objective:

- To observe changes in physical activity in patients with newly initiated therapy with 3D regimen between pre-treatment (baseline) and post-treatment week 12

| |
|---|
| Secondary objectives:<br>• To correlate subjective fatigue (assessed by means of Fatigue Severity Scale [FSS]) and physical activity (measured with an electronic activity tracker) at baseline, during and after 12 weeks of treatment with 3D regimen<br>• To observe the proportion of patients achieving sustained virologic response (SVR12) after treatment with 3D regimen (defined as HCV not detectable, 12 weeks after the last dose of 3D regimen)<br>• To observe sleep efficiency (assessed by means of activity tracker) at baseline, during and after 12 weeks of treatment with 3D regimen |
| **Study Design:**<br>This is an observational, prospective, open label, single-arm, multi-centric, real-life study in HCV-positive patients (genotype 1).<br>The study consists of a treatment preparation phase of 4 weeks to obtain baseline physical activity by use of a wrist-worn activity tracker, a treatment phase (12 weeks) and a follow-up phase (12 weeks) to evaluate treatment response. The activity tracker is worn for 4 weeks before each visit, to obtain stable individual activity data. At every visit, questionnaire-based fatigue is recorded (FSS questionnaire), according to routine clinical care. |

**Population:**

Patients are eligible for observation in this study, if the following applies:

**Inclusion Criteria:**

- Male and female patients aged ≥ 18 years
- Treatment-naïve patients
- Patients monoinfected with CHCV, GT1 (confirmed within the last 36 months or at physicians discretion in case of risk factors)
- Non-cirrhotic patients (based on liver biopsy, fibroscan ≤ 9.6kPa and/or clinical signs)
- The decision to treat with 3D regimen is made by the physician in accordance with the local Swiss product label prior to any decision to approach the patient to participate in this study. [4]
- Patients with debilitating fatigue (FSS ≥ 4)
- Patients willing to participate in the study, and willing to wear an activity tracker

**Exclusion Criteria:**

- Patients with sources of fatigue other than HCV (especially, severe depression (Annex 4), cancer and hormonal disorders causing clinically significant fatigue)
- Patients with conditions that do not allow to adhere to protocol and use of the device at investigator's discretion
- Patients being wheelchair dependent

| |
|---|
| **Variables:**<br>Primary Variable<br>• Change of mean daytime physical activity between baseline (before treatment start) and post-treatment week 12<br>Secondary Variables<br>• Change of Fatigue Severity Score (FSS) between baseline, during and after 12 weeks of treatment with 3D regimen<br>• Correlation between mean daytime physical activity and FSS at baseline, during and after 12 weeks of treatment with 3D regimen<br>• Proportion of patients achieving SVR12 after treatment with 3D regimen (defined as HCV RNA not detectable 12 weeks after the last actual dose of 3D regimen)<br>• Sleep efficiency at baseline, during and after 12 weeks of treatment with 3D regimen |
| **Data Sources:**<br>Source documents are defined as original documents. The investigator will document patient data, including FSS, in his/her own patient files which will serve as source data for the study. Physical activity and sleep data will be collected by use of wrist-worn activity trackers (ActiGraph GT9X Link) which will be processed by device-specific software (ActiLife 6) to obtain the activity and sleep-related variables for analysis (Annex 1). All data collected by the activity tracker will be analyzed by the biostatistician at the end of the study. The data will not be seen by the investigators and patients at any time during data collection (between visit 1 and visit 5 (Figure 1: Study Activities). |
| **Study Size:**<br>Approximately 100 patients in Switzerland will be enrolled. |
| **Data Analysis:**<br>All data will be analyzed by descriptive statistical methods. The correlation between physical |

| |
|---|
| activity (assessed by the activity tracker) and the fatigue score (assessed by FSS) by visit, and the changes between baseline and the respective follow-up visits will be investigated by Spearman's rank correlation coefficient, supported by subgroup analyses. Two-sided 95% confidence intervals will be provided for primary and secondary endpoints, if applicable.<br><br>Details of planned analyses will be described in a separate statistical analysis plan (SAP). |
| **Milestones:**<br><br>Start of Data Collection: 01 January 2017<br><br>Last Patient first Visit: 31 December 2017<br><br>Final Report of Study Results: 30 June 2019 |

# 5.0 Amendments and Updates

None

# 6.0 Milestones

Major study milestones and their planned dates are as follows:

| | |
|---|---|
| Start of Data Collection: | 01 January 2017 |
| Last Patient first Visit | 31 December 2017 |
| Final Report of Study Results: | 30 June 2019 |

# 7.0 Background and Rationale

**Background**

Hepatitis C virus (HCV) infection is one of the most common liver diseases worldwide. Approximately 2% of the global population suffer from chronic HCV infection. In the majority of cases, HCV infection leads to chronic hepatitis [5]. While several genotypes of HCV exist, genotype 1 is the most common genotype in Europe, comprising the majority of infections [6]. HCV replicates as well within extrahepatic tissues and causes a wide range of extrahepatic manifestations. In fact, 40-74% of patients infected with HCV develop at least one extrahepatic manifestation during the course of their disease [7]. Over the recent years, studies have demonstrated that HCV infection can directly cause cognitive impairment, even in the absence of liver cirrhosis [8, 9]: The biological effect of HCV on the brain may result from viral penetration of the central nervous system (CNS) and/or central neuroinflammation leading to alterations in cerebral metabolism, immune activation, and neurotransmission [10, 11].

There are several potential reasons for the high prevalence of neuropsychological disorders in patients with HCV: Individuals with a pre-existing mental illness have been hypothesized to be more likely to contract HCV, or to abuse psychoactive substances affecting cognitive performance [12, 13]. Another predisposing factor may be pharmaceutical HCV treatment, which can trigger neuropsychological disorders [14]. However, an increasing body of evidence suggests a direct

biological effect of HCV infection resulting in brain dysfunction, which may improve following eradication of the virus [10, 11]. Finally, the hepatic function impairment (cirrhosis) may lead to minimal or overt encephalopathies with a broad range of neuropsychological symptoms [15].

Clinically, HCV infection is strongly associated with multiple psychiatric comorbidities. Fatigue, i.e. sensations of both mental and physical exhaustion [16], is the most common symptom reported by patients with HCV [17, 18], which paradoxically may occur as well in conjunction with sleep disturbances, such as insomnia [19]. Fatigue can range from mild to severe and can affect every area of life. Conclusively, American and European guidelines (American Association for the Study of Liver Diseases [AASLD], European Association for the Study of the Liver [EASL]) rank the treatment of HCV-related debilitating fatigue as a high priority [20, 21]. Importantly, this symptom is difficult to quantify objectively and occurs independently from severe liver damage. In addition, there is a lack of controlled studies investigating fatigue in HCV patients [22]. Specifically on the effect of 3D regimen, there is a data gap on the association of treatment success and the improvement of fatigue.

Therapy of HCV has seen huge progress in recent years. Viekirax® (ombitasvir/ paritaprevir/ ritonavir tablets) is indicated in combination with Exviera® (dasabuvir tablets) with or without Ribavirin (RBV) for the treatment of chronic hepatitis C in adults, genotype 1. Combination treatment (3D regimen) combines three different antiviral mode of action and has been shown to result in sustained virologic response (SVR) rates higher than 95 percent [23-25].

**Rationale**

Data demonstrating a longitudinal change of debilitating physical fatigue, along with concurrently increased daytime physical activity upon treatment with 3D regimen are missing to date and are highly anticipated by the Swiss scientific HCV community.

Furthermore, this study attempts to support the widely discussed Swiss Hepatitis Strategy, which seeks for the elimination of viral hepatitis in Switzerland within the next 15 years by creating awareness for patients with extrahepatic manifestations [3].

# 8.0 Research Question and Objectives

## 8.1 Research Question

Does successful treatment with 3D regimen increase total daytime physical activity and reduce fatigue in HCV-positive patients?

## 8.2 Objectives

### 8.2.1 Primary Objective

- To observe changes in physical activity in patients with newly initiated therapy with 3D regimen between baseline (before treatment start) and post-treatment week 12

### 8.2.2 Secondary Objectives

- To correlate subjective fatigue (assessed by means of FSS) and physical activity (measured with an electronic activity tracker) at baseline, during and after 12 weeks of treatment with 3D regimen
- To observe the proportion of patients achieving sustained virologic response (SVR12) after treatment with 3D regimen (defined as HCV RNA not detectable, 12 weeks after the last dose of 3D regimen)
- To observe sleep efficiency (assessed by means of activity tracker) at baseline, during and after 12 weeks of treatment with 3D regimen

# 9.0 Research Methods

## 9.1 Study Design

This prospective, multicenter, post-marketing observational study (PMOS) will be conducted with a single arm design on HCV patients receiving 3D regimen according to routine clinical practice. This study will be conducted in the main Swiss HCV competence centers.

The decision of the treatment regimen is at the discretion of the physician in accordance with local clinical practice and label; it is made independently from this observational study, and precedes the decision to offer the patient the opportunity to participate in this study.

Adult patients diagnosed with HCV, for which the decision to treat with 3D regimen has been made, will be offered the opportunity to participate in this study during a routine clinical visit at the participating sites.

After written informed consent has been obtained, patient data including demographic data, HCV disease characteristics (including questionnaire-assessed fatigue), comorbidities, concomitant medication, and treatment details as recorded in the patient's medical records (source documentation) will be documented in the case report form (CRF).

Follow-up visits, treatment, procedures and diagnostic methods will follow physicians' routine clinical practice. Patients will be observed for approximately 28 weeks. No patient identifiable information will be captured; a unique patient number will be specified in the CRF.

The study activities to be conducted are outlined in the schematic presented below.

## Figure 1: Study Activities

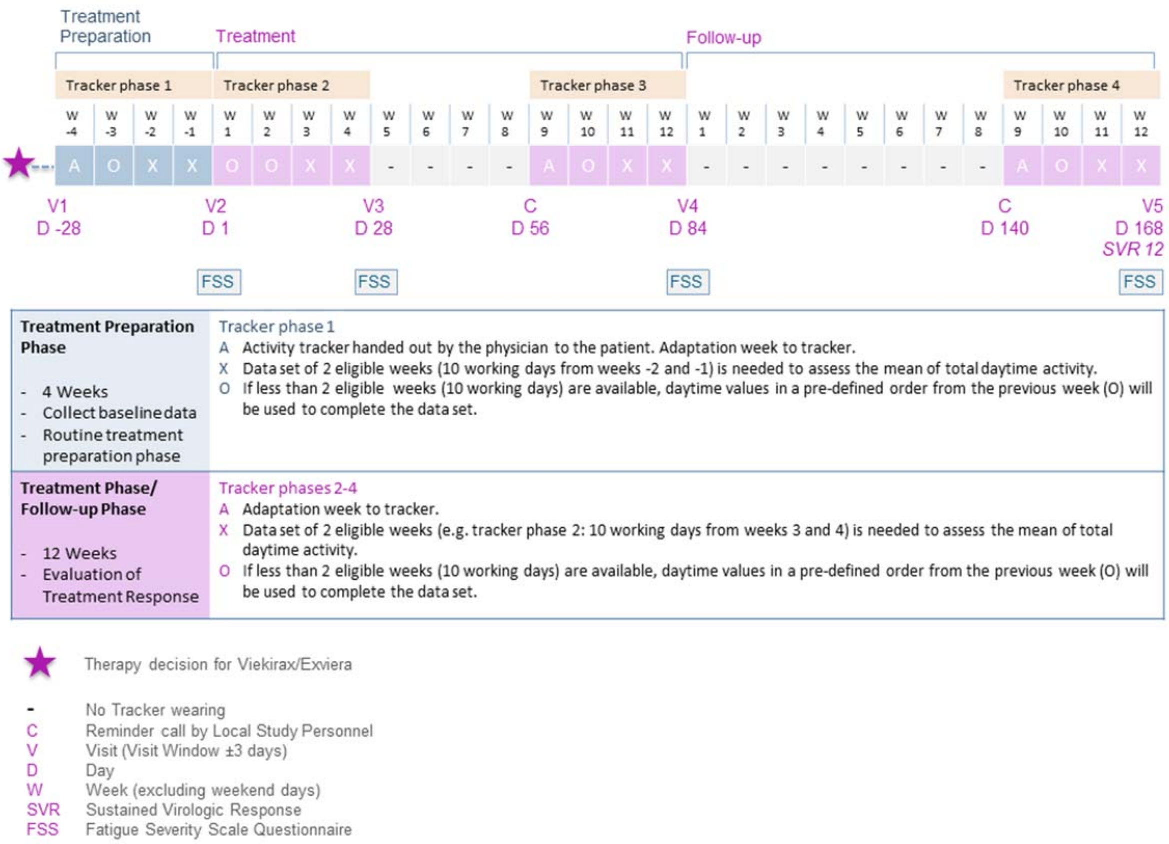


### 9.1.1 Description of Activities

Patients will be screened to ensure they meet the inclusion criteria. All patients will be required to sign an informed consent to use and disclose personal health information. By signing this consent, patients agree to release their information to AbbVie. No study related activity will be performed prior to obtaining written informed consent.

When the investigator has determined that the patient is eligible for participation, and the patient has agreed to be included in this observational study, the patient will be handed out his personal activity tracker (ActiGraph GT9X Link [26]) (Figure 1) and will be instructed in its use. The patient will be advised to report any potential side effects related to the activity tracker, such as possible discomfort wearing the device around the clock or possible skin rash or skin irritation, to the study personnel. ActiGraph GT9X Link has a data storage capacity of 240 days/4GB. Therefore, the patient will keep the tracker for the whole duration of the study (196 days). The patients will be requested to start wearing their activity trackers on the non-dominant arm constantly for 8 weeks between visit 1 and visit 3 and for 4 weeks preceding visits 4 and 5. Everytime the patient has to put on his tracker, he will receive a reminder call from a local study personnel to increase adherence. At the last visit, the investigator will collect the tracker and deliver it to the biostatistician which in turn transfers the activity data to a post-processed environment (ActiLife 6 [27]) (Figure 1).

If the investigator sees the medical indication to initiate treatment with 3D regimen before the patient has worn the activity tracker for at least 4 weeks during the treatment preparation phase, or if 3D regimen is discontinued at the investigator's discretion, the patient will discontinue study participation. ActiGraph GT9X Link comes with a wear time sensor that automatically detects if a wrist worn device has been removed. Thus, patient compliance to the tracker will be measured by this wear time sensor. If values are missing for a total of ≥ 2 hours during daytime, this day will be declared as non-eligible. In order to complete the data set of 2 weeks (10 working days) any non-eligible day will be replaced by daytime values in a pre-defined order, starting with the most recent day of the respective aforegoing week (Figure 1). Weekend days are excluded from analysis.

## Health Outcomes Assessment

### Activity Tracker: ActiGraph GT9X Link (Annex 1) [26]

The ActiGraph GT9X Link has been classified as Class I medical device within the European Union and allows to deliver valuable information about movement, rotation, and body position.

There have been already published several examples of the use of ActiGraph devices in research studies and clinical trials involving physical activity, energy expenditure, and sleep/wake behavior and their relationships to a wide range of health conditions including HCV, obesity, diabetes, cancer, CNS disorders, cardiovascular disease and sleep disorders. [28-37].

The collected activity data can be utilized in a post-processed environment (ActiLife 6). Data is automatically collected from all on-board sensors in raw data format (Annex 1).

After a profound training on the tracker (activity monitor, software ActiLife 6) the local study personnel will be in charge to help and instruct patients correctly in the use and requirements to stay in the study, as well as to assist in the case of tracker-related questions that may arise. Furthermore, a detailed patient information brochure will give useful information about the tracker handling to the patient.

Questionnaire

Patients will be asked to complete the Fatigue Severity Scale (FSS) before treatment start and at each visit as part of routine practice. The FSS is a nine-item questionnaire assessing the functional impact of fatigue during the past two weeks on multiple life domains using scales from 1 (strongly disagree) to 7 (strongly agree). The fatigue score is the mean score of the 9 items. Clinically significant fatigue is usually defined as score equal or above “4” [38, 39]. The routinely assessed FSS score to be used within this study should not include questionnaire results older than 3 months before visit 1. Patients with an average FSS score < 4 at visit 2 will discontinue study participation.

### 9.1.2 Description of Visits

| Activity | Treatment Preparation | Treatment Phase | | | | Follow up | |
|---|---|---|---|---|---|---|---|
| | V1 | V2 | V3 | Phone Call | V4 | Phone Call | V5 |
| | Day -28 | Day 1 | Day 28 | Day 56 | Day 84 | Day 140 | Day 168 SVR12 |
| **Routinely assessed data required for this study** | | | | | | | |
| Patient information and informed consent | X | | | | | | |
| Inclusion/Exclusion Criteria check (Patient Selection) | X | | | | | | |
| Demographics: Year of birth, ethnicity, sex | X | | | | | | |
| HCV History (first diagnosis, date and source of infection, fibrosis stage) | X | | | | | | |
| Medical History (including extrahepatic manifestation) | X | | | | | | |
| HCV Viral load | X | X | X | | X | | X |
| Start the medication regimen | | X | | | | | |
| Adverse events | | X[1] | X | | X | | X[1] |
| Concomitant medication | X | X | X | | X | | X |
| Explain function and wearing of activity tracker | X | | | | | | |
| Handing out the activity tracker | X | | | | | | |
| Investigation of handling with the activity tracker | | X | X | | X | | X |
| Reminder Call to start new cycle of wearing the activity tracker | | | | X | | X | |
| Collect activity tracker | | | | | | | X |
| FSS Questionnaire[2] | X | X | X | | X | | X |

| | | | | | | | |
|---|---|---|---|---|---|---|---|
| **Additional data recorded in the CRF if routinely available** | | | | | | | |
| Physical Exam | X | | | | X | | X |
| Vital signs (blood pressure, pulse, weight, height and body temp) | X | X | X | | X | | X |
| Smoking and Alcohol status | X | | | | | | X |
| Laboratory | X | X | X | | X | | X |
| Treatment documentation (compliance) | | | X | | X | | |
| 1: Capturing Adverse Events and Serious Adverse Events begins with the signature of the informed consent and stops 30 days after the last intake of the 3D regimen.<br>2: To be filled out at every visit by the patient himself and without any prior influence by the study personal. | | | | | | | |

### 9.1.3 Product Supply

Commercially available 3D regimen will be used as per routine clinical practice and local label/guidelines. AbbVie will not provide the medication for this study.

### 9.1.4 Termination Criteria

A patient may withdraw from the study at any time. The investigator may discontinue any patient's participation for any reason. AbbVie AG may interrupt/stop the study at any time for any reason.

If a patient withdraws or is lost to follow-up, the reason for withdrawal will be recorded in the CRF if voluntarily shared by the patient. In case of study termination, the investigator must ensure that the patient returns the activity tracker, and that no subsequent activity tracker will be shipped to the patient.

Specifically, the following termination criteria apply:

- Patients not eligible for treatment with 3D regimen in accordance with the Swiss product label and according to the investigator's judgment will discontinue study participation.
- Patients who discontinue treatment with 3D regimen or who do not wear the activity tracker for at least 3 weeks before each visit will be withdrawn from the study. In case the decision to discontinue treatment with 3D regimen was taken before or during a routine clinic visit, this visit will be considered the last study visit. The last dose of treatment of 3D regimen will be recorded in the CRF.
- Patients with an average FSS score < 4 at visit 2 will discontinue the study.

Following discontinuation of the study, the patient will be treated in accordance with the investigator's best clinical judgment.

## 9.2 Setting

### 9.2.1 Target Population

Patients are eligible for observation in this study, if the following applies:

**Inclusion Criteria:**

- Male and female patients aged ≥ 18 years
- Treatment-naïve patients
- Patients monoinfected with CHCV, GT1 (confirmed within the last 36 months or at physicians discretion in case of risk factors)
- Non-cirrhotic patients (based on liver biopsy, fibroscan ≤ 9.6kPa and/or clinical signs)
- The decision to treat with 3D regimen is made by the physician in accordance with the local Swiss product label prior to any decision to approach the patient to participate in this study.
- Patients with debilitating fatigue (FSS ≥ 4)

- Patients willing to participate in the study, and willing to wear an activity tracker

**Exclusion Criteria:**

- Patients with sources of fatigue other than HCV (especially, severe depression (Annex 4), cancer and hormonal disorders causing clinically significant fatigue)
- Patients with conditions that do not allow to adhere to protocol and use of the device at investigator's discretion
- Patients being wheelchair dependent

#### 9.2.2 Study Duration

This is a prospective, observational study. The enrollment period will be approximately 12 months and the duration of the observational period will be approximately 28 weeks for each patient. Patients may discontinue treatment or the study at any time without prejudice.

#### 9.2.3 Investigator Selection Criteria

The sites will be selected based on the availability of patient population targeted for this study. The selected investigators (qualified by training and experience) and study sites (with proven experience and competence in treating HCV patients) will have the ability to appropriately conduct this observational study in accordance with applicable legal and regulatory requirements.

## 9.3 Variables

### 9.3.1 Primary Variable

- Change of mean daytime physical activity between baseline (before treatment start) and post-treatment week 12

### 9.3.2 Secondary Variables

- Change of Fatigue Severity Score (FSS) between baseline, during and after 12 weeks of treatment with 3D regimen
- Correlation between mean daytime physical activity and FSS at baseline, during and after 12 weeks of treatment with 3D regimen
- Proportion of patients achieving SVR12 after treatment with 3D regimen (defined as HCV RNA not detectable 12 weeks after the last actual dose of 3D regimen)
- Sleep efficiency at baseline, during and after 12 weeks of treatment with 3D regimen

## 9.4 Data Sources

Source documents are defined as original documents. The investigator will document patient data, including FSS, in his/her own patient files which will serve as source data for the study. Physical activity and sleep data will be collected by use of wrist-worn activity trackers (ActiGraph GT9X Link) which will be processed by device-specific software to obtain the physical activity and sleep-related variables for analysis.

All data collected by the activity tracker will be analyzed by the biostatistician at the end of the study. The data will not be seen by the investigators and patients at any time during data collection (between visit 1 and visit 5 (Figure 1: Study Activities).

## 9.5 Study Size

Observational research is exploratory in nature and there are no pre-specified hypotheses to be tested. Hence, there is no need for power calculation. The primary endpoint in this study is derived from an activity tracking device.

In the literature, no data on actigraphy in HCV patients suffering from fatigue are available that would allow statistical sample size calculation focusing on the precision of estimation of the primary endpoint.

On the other hand, the patient selection criteria only allow recruitment of a restricted number of patients in a justifiable period of time. We assume that a total of 100 patients are sufficient to provide stable results regarding investigation of the study objectives.

The planned study size of 100 will have 80% power to detect a change from baseline of the effect size 0.29 using a two-sided one-sample t-test with a significance level of 5%.

## 9.6 Data Management

Data for this study will be recorded in English by each participating center on case report forms (CRFs). Examinations, diagnostic measures, laboratory assessments, findings and observations routinely performed in this cohort, will be transcribed by the investigator or designee from the source documents into the CRF. Only data specified in the observational plan will be entered into the CRF. For each enrolled patient, the investigator or designee will create a new patient file in the CRF.

The investigator or designee will deliver the activity tracker to the study designated biostatistician following the last scheduled patient visit (Table 1). Activity tracker collected data will then be transferred to the data processing software ActiLife 6 [27].

Besides the tracker data, CRF data will be evaluated by a study designated biostatistician.

## 9.7 Data Analysis

All data will be analyzed by descriptive statistical methods.

Quantitative data (e.g. the primary variable) will be analyzed by the statistical parameters valid N, missing N, mean, standard deviation (SD), and selected quantiles: minimum (0%), lower quartile (25%), median (50%), upper quartile (75%), and maximum (100%). If indicated by the data, an additional frequency distribution will be supplied after appropriate grouping of data.

Qualitative (e.g. achievement of SVR12 (yes/no)) and categorical variables (e.g. individual items of the FSS) will be presented by (absolute and relative) frequency distributions.

The primary variable will be tested using the one-sample t-test (two-sided) on the 5% significance level.

Two-sided 95% confidence intervals will be provided for mean daytime physical activity, FSS and sleep efficiency by visit and for the changes between start of treatment and the respective follow-up visits. A 95% confidence interval for the proportion of patients achieving SVR12 will also be calculated.

The correlation between the physical activity (assessed by the activity tracker) and the fatigue score (assessed by FSS) will be investigated by visit and for the changes between baseline and follow-up visits, using Spearman's rank correlation coefficient.

Subgroup analyses will be provided for patients who achieve / do not achieve SVR12 after treatment with 3D regimen.

All statistical analyses will be carried out by means of the SAS® package (version 9.2 or higher).

Details of planned analyses will be described in a separate statistical analysis plan (SAP).

## 9.8 Quality Control

The sites will be instructed in the observational plan, the handling of the CRF and activity tracker, and the requirement to maintain source documents for each patient in the study (see Section 9.4).

Follow-up on CRF data for medical plausibility will be done by AbbVie personnel (or their representatives). The investigator of each site will finally review the CRFs for completeness and accuracy of available data and provide his or her signature and date to CRFs as evidence thereof.

Continuous monitoring of the study and frequent site telephone contacts will be done by AbbVie or a Contract Research Organization (CRO) working on behalf of AbbVie.

Patients will not be compensated for their participation in this PMOS study. Participating physicians will be offered compensation by AbbVie for compiling and submitting clinical information for use in this PMOS study according to the regulatory requirements in Switzerland. In case of inconsistent data entry, the physician will be contacted during query management to complete data of concerned visits. Without complete documentation only partial payment of visits will be performed. Amount of payment per Visit will be documented in contracts between participating site and AbbVie.

## 9.9 Limitations of the Research Methods

The limitations of observational studies, such as uncontrolled confounding by lack of randomization, and difficulties to clearly interpret treatment effects in the context of missing data are well known. Their validity can be increased by accurate outcome measurements, documentation of the most common confounders, sufficient length of follow-up and by activities to obtain complete recording of available data as well as by searches for missing key data.

The most important outcome measure in this study is the measurement of physical activity by the ActiGraph GT9X Link. Failure in wearing the device or misuse may lead to erroneous estimation of the real physical activity pattern of the respective patient.

### 9.10 Other Aspects

N/A

## 10.0 Protection of Human Subjects

This observational study will be run in compliance with local laws and regulations. Notification/submission to the responsible regulatory authorities, Ethics Committee (EC) and/or Competent Authorities (CAs) will be done as required by local laws and regulations.

The investigator is responsible to ensure that written informed consent to use and/or disclose patients' anonymized health data will be obtained prior to patient inclusion.

To maintain patient confidentiality, no demographic data that can identify the patient will be collected (e.g. initials, date of birth) - only the patient age will be collected. In order to protect patient identity, a unique number will be assigned to each patient and related study records.

The study will be entirely financed by AbbVie AG. The patient will not be paid for his participation in the study.

## 11.0 Management and Reporting of Complaints

A Complaint is any written, electronic, or oral communication that alleges deficiencies related to the physical characteristics, identity, quality, purity, potency, durability, reliability, safety, effectiveness, or performance of a product/device after it is released for distribution.

Complaints associated with any component of this product/device must be reported to the Sponsor (Section 11.2.2). For adverse events, please refer to Sections 11.1.1 through 11.1.7. For product complaints, please refer to Section 11.2.

## 11.1 Medical Complaints

### 11.1.1 Adverse Event Definition and Serious Adverse Event Categories

An adverse event (AE) is defined as any untoward medical occurrence in a patient, which does not necessarily have a causal relationship with their treatment. An AE can therefore be any unfavorable and unintended sign (including an abnormal laboratory finding), symptom, or disease temporally associated with the use of a medicinal product, whether or not the event is considered causally related to the use of the product.

Such an event can result from use of the drug as stipulated in the labeling, as well as from accidental or intentional overdose, drug abuse, or drug withdrawal. Any worsening of a pre-existing condition or illness is considered an AE. An AE can also derive from the medical device ActiGraph GT9X Link, which is used in this study. Minimal risks related to the Activity Tracker can be possible discomfort wearing the device around the clock and possible skin rash or skin irritation caused by the device.

Patient reported FSS questionnaire data are not considered a potential source of adverse reactions. However, participating sites should review the questionnaire data and if a possible product-related event (including a suspected adverse reaction) is noted, the Health Care Professional must determine whether the event is related to an AbbVie authorized product and if so, it should be reported to AbbVie. If an increase in the FSS score by $\geq 1$ point is observed, the investigator must report this increased fatigue as an AE to AbbVie.

Patient derived activity tracker data are not considered a potential source of adverse reactions. Physicians are not able to see tracker data at any time. Thus, if a possible product-related event (including a suspected adverse reaction) is noted, activity tracker derived mean values of interest will be reported exclusively at the end of the study by the bio-statistician to the AbbVie PV team.

If an AE meets any of the following criteria, it is considered a serious adverse event (SAE):

| | |
|---|---|
| **Death of Patient:** | An event that results in the death of a patient. |
| **Life-Threatening:** | An event that, in the opinion of the investigator, would have resulted in immediate fatality if medical intervention had not been taken. This does not include an event that would have been fatal if it had occurred in a more severe form. |
| **Hospitalization:** | An event that results in an admission to the hospital for any length of time. This does not include an emergency room visit or admission to an outpatient facility. |
| **Prolongation of Hospitalization:** | An event that occurs while the study patient is hospitalized and prolongs the patient's hospital stay. |
| **Congenital Anomaly:** | An anomaly detected at or after birth or any anomaly that results in fetal loss. |
| **Persistent or Significant Disability/Incapacity:** | An event that results in a condition that substantially interferes with the activities of daily living of a study patient. Disability is not intended to include experiences of relatively minor medical significance such as headache, nausea, vomiting, diarrhea, influenza, and accidental trauma (e.g., sprained ankle). |
| **Important Medical Event Requiring Medical or Surgical Intervention to Prevent Serious Outcome:** | An important medical event that may not be immediately life-threatening or result in death or hospitalization, but based on medical judgment may jeopardize the patient and may require medical or surgical intervention to prevent any of the outcomes listed above (i.e., death of patient, life threatening, hospitalization, prolongation of hospitalization, congenital anomaly, or persistent or significant disability/incapacity). Additionally, any elective or |

spontaneous abortion or stillbirth is considered an important medical event. Examples of such events include allergic bronchospasm requiring intensive treatment in an emergency room or at home, blood dyscrasias or convulsions that do not result in inpatient hospitalization, or the development of drug dependency or drug abuse.

### 11.1.2 Severity

The following definitions will be used to rate the severity for any AE being collected as an endpoint/data point in the study and for all SAEs.

| | |
|---|---|
| **Mild:** | The AE is transient and easily tolerated by the patient. |
| **Moderate:** | The AE causes the patient discomfort and interrupts the patient's usual activities. |
| **Severe:** | The AE causes considerable interference with the patient's usual activities and may be incapacitating or life threatening. |

### 11.1.3 Relationship to Pharmaceutical Product

The following definitions will be used to assess the relationship of the adverse event to the use of product:

| | |
|---|---|
| **Reasonable Possibility** | An AE where there is evidence to suggest a causal relationship between the product and the adverse event. |
| **No Reasonable Possibility** | An AE where there is no evidence to suggest a causal |

relationship between the product and the adverse event.

If no reasonable possibility of being related to product is given, an alternate etiology must be provided for the AE.

### 11.1.4 Serious-, non Serious Adverse Event and Pregnancy Collection Period

SAE, AE and pregnancy will be reported to AbbVie from the time the physician obtains the patient's authorization to use and disclose information (or the patient's informed consent) until 30 days or 5 half-lives following the intake of the last dose of physician-prescribed treatment, which ever period is longer.

### 11.1.5 Adverse Event reporting

For events from patients using and AbbVie product - notify the AbbVie contact person identified below within 5 business days of the physician becoming aware of the event.

Pharmacovigilance AbbVie AG Schweiz:
Phone: +41 41 399 16 09
Fax: +41 41 399 16 67
pharmacovigilance.ch@abbvie.com

### 11.1.6 Serious Adverse Event Reporting

For events from patients using and AbbVie product - notify the AbbVie contact person identified below within 24 hours of the physician becoming aware of the event.

Pharmacovigilance AbbVie AG Schweiz:
Phone: +41 41 399 16 09
Fax: +41 41 399 16 67
pharmacovigilance.ch@abbvie.com

### 11.1.7 Pregnancy Reporting

In the event of a pregnancy occurrence in the female patient or in the female partner of a male patient, the physician will notify AbbVie contact person identified in Section 11.1.6 within 24 hours of the physician becoming aware of the pregnancy.

## 11.2 Product Complaint

### 11.2.1 Definition

A Product Complaint is any Complaint (see Section 11.0 for the definition) related to the biologic or drug component of the product. A Product Complaint can also derive from the medical device ActiGraph GT9X Link, which is used in this study.

For a product this may include, but is not limited to, damaged/broken product or packaging, product appearance whose color/markings do not match the labeling, labeling discrepancies/inadequacies in the labeling/instructions (example: printing illegible), missing components/product, or packaging issues.

Any information available to help in the determination of causality to the events outlined directly above should be captured.

### 11.2.2 Reporting

Product Complaints concerning the product/ device must be reported to the Sponsor within 24 hours of the study site's knowledge of the event via local Product Complaint reporting practices. Product Complaints occurring during the study will be followed-up to a satisfactory conclusion. All follow-up information is to be reported to the Sponsor (or an authorized representative) and documented in source as required by the Sponsor. Product Complaints associated with adverse events will be reported in the study summary. All other complaints will be monitored on an ongoing basis.

Product complaints involving a non-Sponsor product / device should be reported to the identified contact or manufacturer, as necessary per local regulations.

Product Complaints may require return of the product/ device with the alleged complaint condition. In instances where a return is requested, every effort should be made by the investigator to return the product within 30 days. If returns cannot be accommodated within 30 days, the site will need to provide justification and an estimated date of return.

The description of the complaint is important for AbbVie in order to enable AbbVie to investigate and determine if any corrective actions are required.

## 12.0 Plans for Disseminating and Communicating Study Results

At the end of this observational study, a report will be written by AbbVie or a CRO working on behalf of AbbVie. The required standard study report template will be followed. This report will contain a description of the objectives of the study, the methodology and its results and conclusions. The completed CRFs, [patient questionnaires, interim assessments], the final study output and study report are the confidential property of AbbVie and may not be released to unauthorized people in any form (publications or presentations) without express written approval from AbbVie. The study results will be submitted to local authorities per local laws and regulations.

The results of this study will be made publicly available on one of the primary registries in the World Health Organization (WHO) Registry Journal Network which meet the requirements of the International Committee of Medical Editors (ICMJE) and through scientific publications. Authorship will be in line with ICMJE' authorship requirements [40].

## 13.0 References

1. Negro, F., et al., *Extrahepatic morbidity and mortality of chronic hepatitis C.* Gastroenterology, 2015. **149**(6): p. 1345-1360.
2. Swiss Association for the Study of the Liver (SASL), *SASL-SSI Expert Opinion Statement on the Treatment of Chronic Hepatitis C - January 2016 Update*. 2016: Available Online at https://sasl.unibas.ch/6SASLguidelines.php, accessed 9 June 2016.
3. Swiss Experts in Viral Hepatitis (SEVHep), *Swiss National Strategy to Combat Chronic Viral Hepatitis.* 2015: Available online, at www.hepatitis-schweiz.ch/files/Dokumente/Process_Paper_2nd_version_final.pdf, accessed 9 June 2016.
4. *Summary of Product Characteristics for Viekirax® (ombitasvir/paritaprevir/ritonavir) and Exviera® (dasabuvir); February 2016 (Exviera), March 2016 (Viekirax). Available online, at www.swissmedicinfo.ch.*
5. Lavanchy, D., *The global burden of hepatitis C.* Liver Int, 2009. **29 Suppl 1**: p. 74-81.
6. Messina, J.P., et al., *Global distribution and prevalence of hepatitis C virus genotypes.* Hepatology, 2015. **61**(1): p. 77-87.
7. Ahmed, N.H. and M.H. Emara, *Extrahepatic Manifestations of Hepatitis C Virus: An Extending List.* Afro-Egyptian Journal of Infectious and Endemic Diseases, 2012: p. 36.
8. Kraus, M.R., et al., *Improvement of neurocognitive function in responders to an antiviral therapy for chronic hepatitis C.* Hepatology, 2013. **58**(2): p. 497-504.
9. Pattullo, V., et al., *Influence of hepatitis C virus on neurocognitive function in patients free from other risk factors: validation from therapeutic outcomes.* Liver Int, 2011. **31**(7): p. 1028-38.
10. Bajaj, J.S., *The role of microbiota in hepatic encephalopathy.* Gut Microbes, 2014. **5**(3): p. 397-403.
11. Wilkinson, J., et al., *Activation of brain macrophages/microglia cells in hepatitis C infection.* Gut, 2010. **59**(10): p. 1394-1400.
12. Rosenberg, S.D., et al., *Prevalence of HIV, hepatitis B, and hepatitis C in people with severe mental illness.* American journal of public health, 2001. **91**(1): p. 31.
13. McAndrews, M.P., et al., *Prevalence and significance of neurocognitive dysfunction in hepatitis C in the absence of correlated risk factors.* Hepatology, 2005. **41**(4): p. 801-808.
14. Schaefer, M., et al., *Hepatitis C infection, antiviral treatment and mental health: a European expert consensus statement.* Journal of hepatology, 2012. **57**(6): p. 1379-1390.

15. Poordad, F.F., *Review article: the burden of hepatic encephalopathy.* Aliment Pharmacol Ther, 2007. **25 Suppl 1**: p. 3-9.
16. Monaco, S., et al., *HCV-related nervous system disorders.* Clinical and Developmental Immunology, 2012. **2012**.
17. Lang, C.A., et al., *Symptom prevalence and clustering of symptoms in people living with chronic hepatitis C infection.* Journal of Pain and Symptom Management, 2006. **31**(4): p. 335-344.
18. Poynard, T., et al., *Fatigue in patients with chronic hepatitis C.* Journal of viral hepatitis, 2002. **9**(4): p. 295-303.
19. Carlson, M.D., et al., *Role of Sleep Disturbance in Chronic Hepatitis C Infection.* Current Hepatitis Reports, 2010. **9**(1): p. 25-29.
20. AASLD-IDSA HCV Guidance Panel, *Hepatitis C guidance: AASLD-IDSA recommendations for testing, managing, and treating adults infected with hepatitis C virus.* Hepatology, 2015. **62**(3): p. 932-54.
21. European Association for the Study of the Liver (EASL), *EASL Recommendations on Treatment of Hepatitis C 2015.* J Hepatol, 2015. **63**(1): p. 199-236.
22. Sarkar, S., et al., *Fatigue before, during and after antiviral therapy of chronic hepatitis C: results from the Virahep-C study.* J Hepatol, 2012. **57**(5): p. 946-52.
23. Ferenci, P., et al., *ABT-450/r-ombitasvir and dasabuvir with or without ribavirin for HCV.* N Engl J Med, 2014. **370**(21): p. 1983-92.
24. Feld, J.J., et al., *Treatment of HCV with ABT-450/r-ombitasvir and dasabuvir with ribavirin.* N Engl J Med, 2014. **370**(17): p. 1594-603.
25. Poordad, F., et al., *ABT-450/r-ombitasvir and dasabuvir with ribavirin for hepatitis C with cirrhosis.* N Engl J Med, 2014. **370**(21): p. 1973-82.
26. Actigraph, *ActiGraph GT9X Link User's Manual*. 2014: Available online, at www.actigraphcorp.com/support/downloads, accesssed 9 June 2016.
27. Actigraph, *ActiLife 6 User's Manual*. 2012: Available online, at www.actigraphcorp.com/support/downloads, accessed 9 June 2016.
28. Actigraph, *Actigraph Research database*. Available online, at www.actigraphcorp.com/resources/research-database, accessed 9 June 2016.
29. Heeren, M., et al., *Active at night, sleepy all day–Sleep disturbances in patients with hepatitis C virus infection.* Journal of hepatology, 2014. **60**(4): p. 732-740.
30. Chau, J.Y., et al., *More standing and just as productive: Effects of a sit-stand desk intervention on call center workers' sitting, standing, and productivity at work in the Opt to Stand pilot study.* Preventive Medicine Reports, 2016. **3**: p. 68-74.
31. Pawlowski, C.S., et al. *Children's physical activity behavior during school recess: A case study using GPS, accelerometer, participant observation, and go-along interview*. in *Poster session presented at the congress: International Society of Behavioral Nutrition and Physical Activity, Edinburgh, Scotland*. 2015.

32. Clemente, F.M., et al., *Physical Activity Patterns in University Students: Do They Follow the Public Health Guidelines?* PloS one, 2016. **11**(3): p. e0152516.
33. Wick, K., et al., *Deviation between self-reported and measured occupational physical activity levels in office employees: effects of age and body composition.* International archives of occupational and environmental health, 2015: p. 1-8.
34. Donath, L., et al., *Repetitive Daily Point of Choice Prompts and Occupational Sit-Stand Transfers, Concentration and Neuromuscular Performance in Office Workers: An RCT.* International journal of environmental research and public health, 2015. **12**(4): p. 4340-4353.
35. Burns, R.D., et al., *Establishing school day pedometer step count cut-points using ROC curves in low-income children.* Preventive medicine, 2016. **86**: p. 117-122.
36. Schall, M.C., N.B. Fethke, and H. Chen, *Evaluation of four sensor locations for physical activity assessment.* Applied ergonomics, 2016. **53**: p. 103-109.
37. Slater, J.A., et al., *Assessing sleep using hip and wrist actigraphy.* Sleep and Biological Rhythms, 2015. **13**(2): p. 172-180.
38. Kleinman, L., et al., *Psychometric evaluation of the fatigue severity scale for use in chronic hepatitis C.* Qual Life Res, 2000. **9**(5): p. 499-508.
39. Rosa, K., et al., *Validation of the Fatigue Severity Scale in chronic hepatitis C.* Health and quality of life outcomes, 2014. **12**(1): p. 1.
40. International Committee of Medical Journal Editors, *Defining the role of authors an contributors*. Available at http://www.icmje.org/recommendations/browse/roles-and-responsibilities/defining-the-role-of-authors-and-contributors.html, accessed 19 May 2016.
41. *ICD-10 Version:2016, http://apps.who.int/classifications/icd10/browse/2016/en#/F32.2, accessed 07 July 2016.*

# Annex 1. ActiGraph GT9X Link [26]

The ActiGraph GT9X Link is the most sophisticated activity tracker available from the global leader in actigraphy measurement. The ActiGraph Link combines an extensively validated accelerometry measurement technology with a variety of advanced new features, including:

Wear time sensor: Automatically detects if a wrist worn device has been removed for simplified compliance monitoring and data cleaning.

Programmable display: An LCD window displays date and time, provides optional real-time subject feedback, or can be completely disabled.

The Tracker will be retrieved by the study personel at the timepoint of study termination.

| **Features & Analysis** | **Objective Measures** |
|---|---|
| LCD Screen | Raw Acceleration (G’s) |
| Gyroscope / Magnetometer | Energy Expenditure |
| Heart Rate Monitoring | MET Rates |
| Proximity Detection | Steps Taken |
| Wear Time Sensor | Physical Activity Intensity |
| Daytime Activity | Activity Bouts |
| Basic Sleep Scoring | Sedentary Bouts |
| Inclinometer Graphing | Heart Rate R-R Intervals |
| Data Vault Access | Body Position |
| | Sleep Latency |
| | Total Sleep Time |
| | Sleep Efficiency |

## ActiGraph GT9X Link: Regulatory Requirements

- The ActiGraph Link is a Class I medical device within the European Union that abides by the regulatory requirements listed below:EN60601-1-Medical Device General Safety Requirements
- Part 15.109 (US) – Radiated Emission Limits of Unintentional RadiatorsPart 15.249 (US) – Radiated Emission Limits of Intentional Radiators
- ICES-003 (Canada) – Interference Causing Equipment Standards for a Digital Apparatus.
- ActiGraph is compliant with IEC standards for "Type BF Applied Part"
- ActiGraph Link and ActiGraph wGT3X-BT is water resistant in accordance with IEC 60529 IPX7, or immersion in one (1) meter of water for up to 30 minutes.
- All ActiGraph products are manufactured Lead Free and are RoHS Compliant.

## Specifications

| | |
|---|---|
| Dimensions | 3.5 x 3.5 x 1 cm |
| Weight | 14 grams |
| Sample Rate | 30-100 Hertz |
| Dynamic Range (Primary Accelerometer) | +/- 8G |
| Dynamic Range (Secondary Accelerometer) | +/- 16G |
| Gyroscope Dynamic Range | +/- 2000 deg/sec |
| Magnetometer Dynamic Range | +/- 4800 micro-Tesla |
| Battery Life | 14 days* |
| Data Storage | 240 days / 4 GB |
| Communication | USB, Bluetooth® LE |
| Water Resistance | 1 meter, 30 minutes |
| Wear Location | Wrist, waist |
| Warranty | 1 year |

*Wireless disabled, 30 Hz sample rate, gyro disabled, sleep mode

## Annex 2. Fatigue Severity Scale (FSS) [38]

Below are a series of statements regarding your fatigue. By fatigue we mean a sense of tiredness, lack of energy or total body give-out.
Please read each statement and choose a number from 1 to 7, where # 1 indicates you completely disagree with the statement and # 7 indicates you completely agree.

Please answer these questions as they apply to the past **TWO WEEKS**.

| | Completely Disagree | | | | | | Completely Agree |
|---|---|---|---|---|---|---|---|
| 1. My motivation is lower when I am fatigued | 1 | 2 | 3 | 4 | 5 | 6 | 7 |
| 2. Exercise brings on my fatigue | 1 | 2 | 3 | 4 | 5 | 6 | 7 |
| 3. I am easily fatigued | 1 | 2 | 3 | 4 | 5 | 6 | 7 |
| 4. Fatigue interferes with my physical functioning | 1 | 2 | 3 | 4 | 5 | 6 | 7 |
| 5. Fatigue causes frequent problems for me | 1 | 2 | 3 | 4 | 5 | 6 | 7 |
| 6. My fatigue prevents sustained physical functioning | 1 | 2 | 3 | 4 | 5 | 6 | 7 |
| 7. Fatigue interferes with carrying out certain duties and responsibilities | 1 | 2 | 3 | 4 | 5 | 6 | 7 |
| 8. Fatigue is among my 3 most disabling symptoms | 1 | 2 | 3 | 4 | 5 | 6 | 7 |
| 9. Fatigue interferes with my work, family, or social life | 1 | 2 | 3 | 4 | 5 | 6 | 7 |

# Annex 3. ICD-10 Version:2016 [41]

**F32.2 Severe depressive episode without psychotic symptoms**

An episode of depression in which several of the above symptoms are marked and distressing, typically loss of self-esteem and ideas of worthlessness or guilt. Suicidal thoughts and acts are common and a number of "somatic" symptoms are usually present.

| | |
|---|---|
| Agitated depression<br>Major depression<br>Vital depression | single episode without psychotic symptoms |

**F32.3 Severe depressive episode with psychotic symptoms**

An episode of depression as described in F32.2, but with the presence of hallucinations, delusions, psychomotor retardation, or stupor so severe that ordinary social activities are impossible; there may be danger to life from suicide, dehydration, or starvation. The hallucinations and delusions may or may not be mood-congruent.

Single episodes of:

- major depression with psychotic symptoms
- psychogenic depressive psychosis
- psychotic depression
- reactive depressive psychosis

## Annex 4. List of Protocol Signatories

AbbVie AG (AbbVie)

Post Marketing Observational Study

Protocol P16-272

Quality of Life measurement using wrist actigraphy in HCV genotype 1 infected, treatment naïve patients suffering from fatigue and receiving ombitasvir, paritaprevir, and ritonavir tablets and dasabuvir tablets (Viekirax®/Exviera®; 3D regimen): The HEMATITE Study

**Approved by:**

| Signatory | Date |
|---|---|
| Protocol Author - Ralph Torgler, PhD<br>Medical Manager HCV, AbbVie AG Schweiz | 28/9/2016 |
| Study-Designated Physician - Marianne Saurenmann, MD<br>Clinical Operations Manager, AbbVie AG Schweiz | 28Sep2016 |
| Statistics Representative - Dr. Dieter Schremmer<br>Head of Biostatistics, GKM Gesellschaft für Therapieforschung mbH | |
| Project Director – Fernando Tatsch, MD<br>Medical Affairs TA Head, AbbVie Global Medical Affairs | |

## Annex 4. List of Protocol Signatories

AbbVie AG (AbbVie)

Post Marketing Observational Study

Protocol P16-272

Quality of Life measurement using wrist actigraphy in HCV genotype 1 infected, treatment naïve patients suffering from fatigue and receiving ombitasvir, paritaprevir, and ritonavir tablets and dasabuvir tablets (Viekirax®/Exviera®; 3D regimen): The HEMATITE Study

**Approved by:**

Protocol Author - Ralph Torgler, PhD
Medical Manager HCV, AbbVie AG Schweiz

Date

Study-Designated Physician - Marianne Saurenmann, MD
Clinical Operations Manager, AbbVie AG Schweiz

Date

28/09/2016

Statistics Representative - Dr. Dieter Schremmer
Head of Biostatistics, GKM Gesellschaft für Therapieforschung mbH

Date

Project Director – Fernando Tatsch, MD
Medical Affairs TA Head, AbbVie Global Medical Affairs

Date

## Annex 4. List of Protocol Signatories

AbbVie AG (AbbVie)

Post Marketing Observational Study

Protocol P16-272

Quality of Life measurement using wrist actigraphy in HCV genotype 1 infected, treatment naïve patients suffering from fatigue and receiving ombitasvir, paritaprevir, and ritonavir tablets and dasabuvir tablets (Viekirax®/Exviera®; 3D regimen): The HEMATITE Study

**Approved by:**

Protocol Author - Ralph Torgler, PhD
Medical Manager HCV, AbbVie AG Schweiz

Date

Study-Designated Physician - Marianne Saurenmann, MD
Clinical Operations Manager, AbbVie AG Schweiz

Date

Statistics Representative - Dr. Dieter Schremmer
Head of Biostatistics, GKM Gesellschaft für Therapieforschung mbH

Date

SEPT 28TH 16

Project Director – Fernando Tatsch, MD
Medical Affairs TA Head, AbbVie Global Medical Affairs

Date

## Annex 5. Requirements for non-interventional studies per local laws and regulations

Competent Authority approval ☐

Competent Authority notification ☐

Competent Authority involvement not required ☒

Ethics Committee approval ☒

Ethics Committee notification ☐

Ethics Committee involvement not required ☐

Written Patient Informed Consent required: ☐ No ☒ Yes

Regulatory requirements, other (if applicable):

*Not applicable*

**Bjoern Wolff, PhD, Medical Director**

Signature                                        03-Oct-2016
                                                 Date
